# Endovascular Interventional Radiology of the Urogenital Tract

**DOI:** 10.3390/medicina57030278

**Published:** 2021-03-17

**Authors:** Fabio Pozzi Mucelli, Roberta A. Pozzi Mucelli, Cristina Marrocchio, Saverio Tollot, Maria A. Cova

**Affiliations:** 1 Department of Radiology, ASUGI, Ospedale di Cattinara, 34149 Trieste, Italy; roberta.pozzimucelli@asugi.sanita.fvg.it; 2 Department of Radiology, University of Trieste, ASUGI, Ospedale di Cattinara, 34149 Trieste, Italy; cristinamarrocchio@gmail.com (C.M.); drtollot.saverio@gmail.com (S.T.); m.cova@fmc.units.it (M.A.C.)

**Keywords:** embolization, angiography, urogenital system, kidney, bladder, priapism

## Abstract

Interventional radiology of the male urogenital system includes percutaneous and endovascular procedures, and these last consist mostly of transcatheter arterial embolizations. At the kidney level, arterial embolizations are performed mainly for palliative treatment of parenchymal tumors, for renal traumas and, less frequently, for arteriovenous fistulas and renal aneurysms and pseudoaneurysms. These latter may often require emergency intervention as they can cause renal or peri-renal hematomas or significant hematuria. Transcatheter arterial embolization is also an effective therapy for intractable severe bladder hematuria secondary to a number of neoplastic and inflammatory conditions in the pelvis, including unresectable bladder cancer and radiation-induced or cyclophosphamide-induced hemorrhagic cystitis. Endovascular interventional procedures for the penis are indicated for the treatment of post-traumatic priapism. In this article, we review the main endovascular radiological interventions of the male urogenital system, describing the technical aspects, results, and complications of each procedure at the various anatomical districts.

## 1. Introduction

In the wide variety of procedures that an interventional radiologist performs daily, the male urogenital interventions represent a relatively large amount of activity. Basically, they are divided into “endovascular” and “percutaneous” procedures. In this review, we will focus on the first ones, and in particular the embolization procedures performed at the renal, bladder, and penile level; venous endovascular interventions, such as varicocele sclerotherapy will not be included in this review. In recent years, embolization of the prostatic arteries in benign prostatic hypertrophy has also become very popular, but this topic will be the subject of a separate review.

At the renal level, embolization procedures are performed mainly for lesions affecting the arteries (aneurysms, pseudoaneurysms, and arteriovenous fistulas) or for arterial bleeding, which can occur spontaneously as in angiomyolipoma, or following a trauma. Bladder embolizations are among the most frequently required procedures by urologists and are performed in case of hematuria that cannot be controlled with other therapies in patients with bladder or prostatic tumors or with actinic cystitis [1,2]. In addition, embolization procedures have become the first-choice therapeutic option in the treatment of high-flow priapism in the penis [3].

We will describe the embolization technique and results.

## 2. Technical Aspects of Arterial Embolizations

Embolization means “obliteration of a vessel by introducing into the bloodstream an occlusive agent, as for example a foreign body or sclerosing fluid, which then creates a deliberate interruption of the blood flow, mechanically or creating an intense inflammatory reaction of the vessel wall” [4]. The purpose of embolization is to be as selective as possible, so as to try to limit the infarction of organ parenchyma.

Before the procedure, previous imaging is obtained or reviewed, if already available, in order to plan the intervention. Imaging modalities include CT or MR, with the imaging study chosen according to the kind of lesion to be treated, the presentation of the patient (whether acute or chronic), and the availability of the techniques in each institution. CT is a fast, reliable and readily available modality, especially useful in a trauma setting [5]. However, drawbacks include delivery of ionizing radiations and use of iodinated contrast material. MR does not use ionizing radiations and it should be considered for both treatment planning and follow-up, especially in younger patients.

Procedures are performed in a digital subtraction angiography unit and generally require only local anesthesia. Most transcatheter embolizations are done with a unilateral retrograde percutaneous femoral approach, using a 4–6 FR sheath. Brachial approach can be used in case of difficulty to find a femoral pulse or if disadvantageous anatomy is suspected on a previous CT evaluation, since it often provides a direct route from the common iliac artery to the hypogastric artery and its branches [6,7].

Once the aorta is reached by the diagnostic catheter, an aortogram or a pelvic angiogram is useful to identify renal arteries or internal iliac arteries. Once the origin of these arteries is detected, they can be catheterized with different pre-shaped catheters according to operator preferences, such as a Cobra, a Simmons-type 1 or 2 or a Berenstein catheter, under fluoroscopic guidance. In cases where a distal branch catheterization is needed, microcatheters (2.1 F to 2.7 F) in association with microguidewires (0.014”–0.018”) can be used.

- When occlusion of terminal arteries is required (such as in case of intrarenal aneurysms), coil embolization is the first choice of treatment. Coils are mechanical devices with different shapes (tridimensional, helical, complex), diameters, and lengths. Often, nylon filaments are added to their surface. Coils can be pushable or detachable, furthermore they can be distinguished in microcoils that need to be used with microcatheters and standard coils, which advance in standard 4-5 F catheters. Pushable coils, once released from the catheter, cannot be retrieved, while the latter, if the final position is not the correct one, may be replaced in the catheter, removed, and changed with another coil and detached by using special devices [8]. Another possible option of treatment are glues. N-butyl-cyanoacrylate (NBCA) was the first liquid embolic agent applied in clinical practice. It is a monomer acrylic glue which quickly polymerizes when it comes in contact with ionic media, such as blood, and causes a permanent occlusion, and also generates an acute inflammatory process in the wall, which progresses to chronic in about four weeks [4]. NBCA is radiolucent and therefore it is usually mixed with radiopaque materials such as Lipiodol. This is an oily contrast medium that, when mixed with NBCA, makes it radiopaque and increases its polymerization time. The NBCA:Lipiodol mixing ratio determines the effect on polymerization time and is adjusted depending on the clinical situation [9]. To avoid adherence to the thin catheters that are required for the superselective embolization, NBCA has to be injected through a catheter washed with a 5% dextrose solution and the catheter has to be withdrawn promptly after injection. Moreover, NBCA polymerizes with an exothermic reaction, causing pain to the patient. Glubran 2 is an acrylic glue bearing a CE mark authorized for surgical and endovascular use in neuroradiology and interventional radiology. The comonomer of Glubran 2 comprises a monomer of NBCA and a monomer of metacryloxysulpholane (MS) (owned by GEM Srl). MS allows the monomer of NBCA to polymerize with a lower exothermic reaction (45 °C) and a slightly longer polymerization time [10]. Compared to the monomer NBCA, the Glubran 2 causes less pain to the patient and is associated with a lower risk of adherence of the catheter to the tissue, hence showing a greater ease of use. Differently, acrylic glues, once deposited into the nidus, determine its permanent occlusion and prevent its replenishment through feeding branches.

Further liquid embolizing material is Onyx^®^ [11]. This is a liquid agent with low adhesive capacity, which has a slow polymerization. It is an alcohol-vinyl-ethylene copolymer (EVOH), containing dimethyl sulfoxide (DMSO) and tantalum powder. It is available in two presentations according to its viscosity: Onyx^®^ 18 and Onyx^®^ 34. It is a controllable material, which produces complete filling, is cohesive and non-adhesive [4].

Particulate agents can also be used; most of them are based on polyvinyl alcohol (PVA) particles of different sizes from 50 to 1200 μm [12]. Usually, embolization with PVA particles causes a permanent occlusion of vessels of the size of the particles used. Recent evolution includes new types of microspheres consisting of a biocompatible acrylamide PVA macromer, which show deformable capability and lower tendency to aggregate inside the catheter during injection with lower adverse body reactions. Alternatives are gelatin or fibrin sponge. These materials can be manually reduced to small fragments by the operator and then mixed with contrast media and slowly injected under fluoroscopic control. However, in the last year, biocompatible, hydrophilic, and dry pre-cut cubes of resorbable porcine gelatin packaged in a 10-mL syringe with a standard luer lock tip ready for use are available. They are quite easy to use, but the main limitation is that the effect of this type of embolization is limited to a short period, and after 10–20 days, many of the vessels may be recanalized [8].

- When occlusion of a main artery is necessary, the possible solutions are coils or plugs. Usually standard catheters and coils are sufficient and rarely are microcatheters required.

Plugs are able to obtain a fast occlusion of the target vessel and are made of nitinol with dense mesh, have different shapes and sizes, and are self-expandable. Once released in the vessel, if the deployment is not correct, the device can be recaptured and repositioned. When the plug is in the correct position, it can be detached easily. Compared to coils, their great advantage is that occlusion is obtained with a single device instead of several coils, thus reducing time and costs of the procedure [8]. In selected cases of aneurysms involving the origin of one or more segmental branches, wide neck complex embolization techniques such as “jailing” technique or “coil-through” technique can be attempted. These techniques have been proposed for endovascular treatment of wide neck intracranial aneurysms but they can be applied also for complex renal aneurysms [13,14]. 

At the end of the embolization procedure, a final angiography is performed to confirm adequate occlusion. Sometimes, it may be necessary to perform the embolization also on the contralateral side (i.e., in case of bladder embolization), and it may be done using the same vascular access or through a contralateral approach. 

## 3. Kidney

### 3.1. Renal Artery Aneurysms

Autopsy studies report an incidence of about 0.01% to 0.09% of renal artery aneurysms (RAAs) [15,16]. However, recent imaging studies state the incidence of RAA to be almost 1% [17]. The classification can be based on their etiology, anatomic location, or morphology. Based on this last parameter, the subtypes can be fusiform, saccular, dissecting aneurysms, and microaneurysms. A true aneurysm is made up of all three layers of the vascular wall, while the wall of a pseudoaneurysm is partially made up of the tissues surrounding the vessel. 

**Figure 1:**
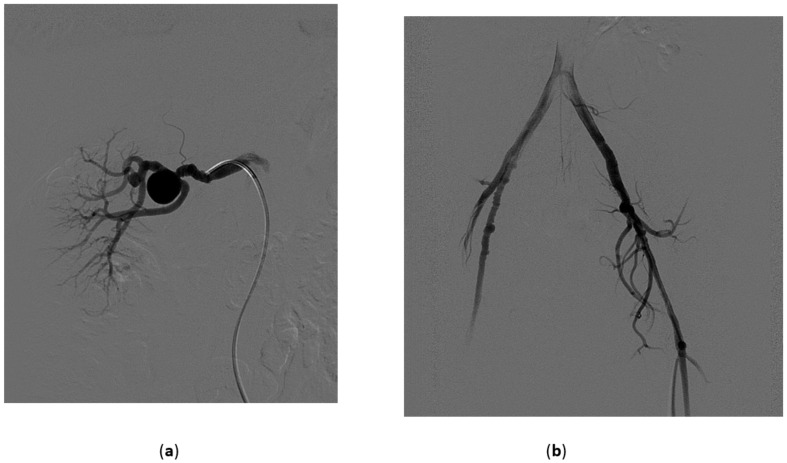

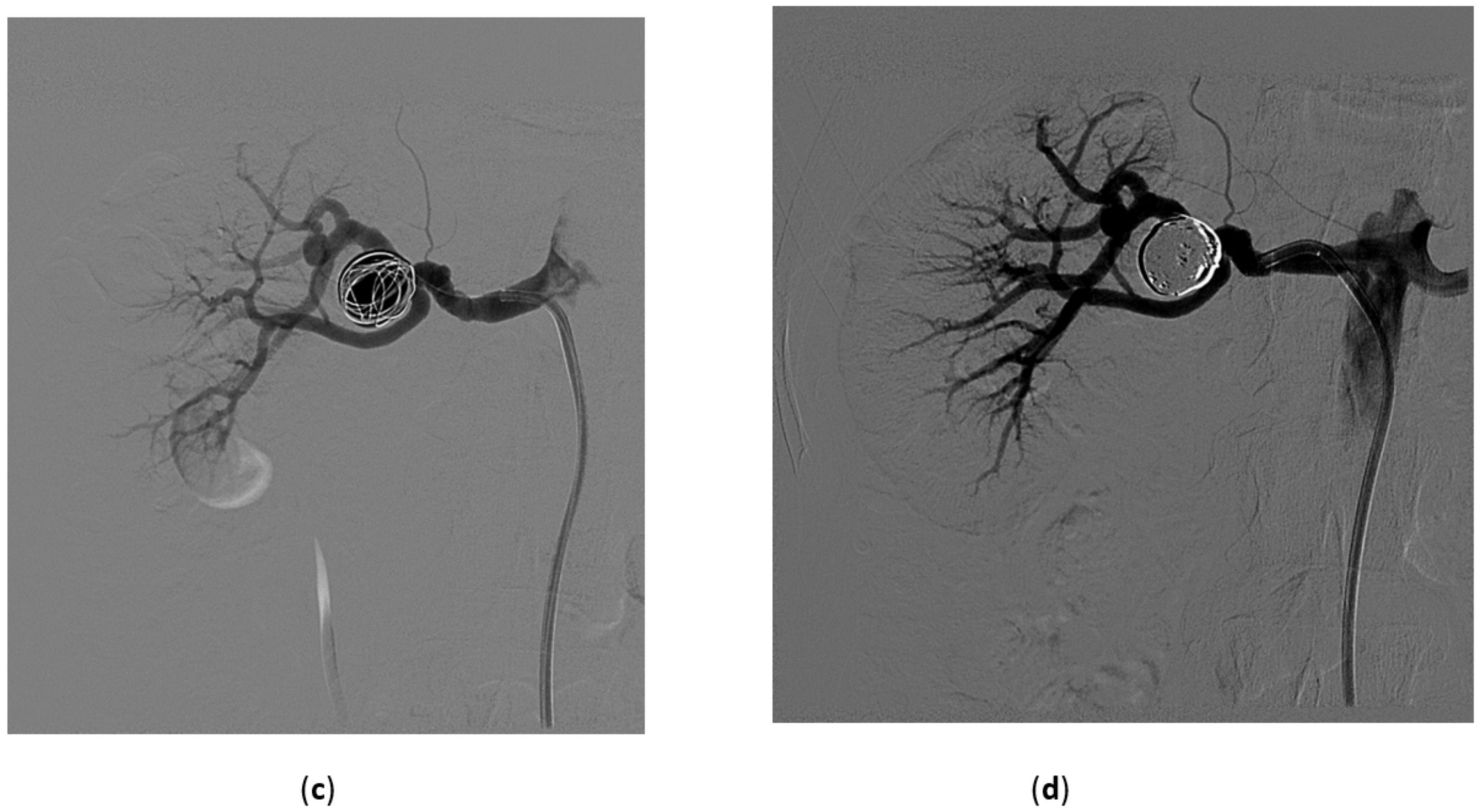
Renal artery aneurysm. 43-year-old female, asymptomatic; at color Doppler Ultrasound (done for other reasons) a 2.5 cm aneurysm of the right renal artery was discovered. (**a**) Preliminary angiogram confirms the aneurysm at the bifurcation of the main trunk. Slight aspects of fibromuscular dysplasia are visible on the distal main trunk. A second small aneurysm is visible on a distal branch; (**b**) An angiogram done on the iliac arteries confirm the diagnosis of fibromuscular dysplasia which is clearly visible on the right external iliac artery; (**c**) Angiogram after deployment of the first detachable microcoil; (**d**) Final angiogram after deployment of 250 cm of coils.

Furthermore, macroaneurysms affecting the renal arteries are more frequently reported in fibromuscular dysplasia (FMD) than in the general population (34%) [18]. FMD is a non-atherosclerotic, non-inflammatory arterial disease affecting more frequently renal and cervical arteries, but other sites may be affected as well (Figure 1). 

Concerning the location, RAAs may be parenchymal or extra-parenchymal. Extra-parenchymal RAAs are seen more often in the mid and distal third of the renal artery and have a tendency to affect the point where the vessel splits. More than 50% of RAAs occur at the bifurcation of the main renal artery [18]. Parenchymal aneurysms can affect proximal lobar arteries as well as deep small arterioles close to the calyces. 

Although in literature it is reported that surgical intervention should be performed at a diameter >2 cm, there is no consensus concerning the treatment of RAAs because there is less data available that analyzes the natural history of these types of aneurysms [19]. Rupture of RAA has a likely incidence of <3% of cases and its prevention should be the primary goal, as this event leads to a mortality rate of approximately 10% [19].

The type of treatment of RAAs depends mainly on the location of the aneurysm.

In case of intrarenal aneurysms, considering that the renal artery is a terminal artery, it makes them quite easy to treat. Coil embolization is the first-choice treatment, although glue could also be used. If aneurysms are very large, they may evolve in an arteriovenous fistula. When this occurs, Amplatzer plugs or detachable coils may be used, to avoid migration of embolic material, with or without adjunctive use of glue or Onyx [11]. 

In case of aneurysms involving the main renal artery, the parent artery needs to be preserved in order to maintain as much renal parenchyma as possible. In some cases that involve the main renal artery, if anatomically feasible, a short stent graft (balloon or self-expandable) can be used [20]. Exclusion of large fusiform aneurysms in branch arteries can be obtained with stent graft placement in the parent artery from which the branch artery arises. Other possible options are coiling of the aneurysm passing with a microcatheter through the struts of a bare stent, or the use of a flow-diverter stent. 

In case of aneurysms at the bifurcation of the main artery, if the aneurysm has a narrow neck, it could be possible to fill it completely with coils preserving the parent artery and allowing the patency of all major renal artery branches (Figure 1).

Endovascular treatment has a low incidence of complications such as infarction due to poor selective embolization or incorrect deployment of embolic material (coils or glue). Other possible complications are thrombosis of the stent graft, failure of exclusion with enlargement of the aneurysm, infection, and flank pain [21].

### 3.2. Renal Artery Pseudoaneurysm (RAP)

A pseudoaneurysm is defined as an injury to one or more layers of the arterial walls. A RAP is an “aneurysm” that develops in a renal artery. The lesion causes a bleeding that can infiltrate between two layers of the arterial wall and thus a hematoma is formed. This is usually limited due to the presence of the surrounding tissue. After the bleeding has stopped, reactive fibrosis occurs and a structure that resembles an aneurysmatic sac is formed. However, even after that, a pseudoaneurysm can re-expand due to a new connection between intra- and extra-luminal space.

A pseudoaneurysm is a serious occurrence because it could turn into a life-threatening condition in case of uncontrolled bleeding if the balance between the intraluminal pressure of the affected artery overcomes the extraluminal tamponade effect [22]. The first description of RAP was done after a partial nephrectomy and reported in 1973 by Rezvani et al. [23]. After that, an increasingly large number of case reports stated that RAP is a possible complication of surgery. Following the introduction of minimally invasive partial nephrectomy (MIPN), the number of RAPs increased even more, as confirmed in the meta-analysis conducted by Jain et al. [24]. This higher incidence may be due to the more complex surgical steps required during MIPN; however, assertions in this comparison are merely speculative, as no randomized trials comparing these surgical techniques are available.

Always according to Jain et al. [24], 97% of the patients present with one of three classic symptoms: gross hematuria, flank pain, and anemia; the latter is the most persistently present as is observed in 87.3% of the cases.

**Figure 2:**
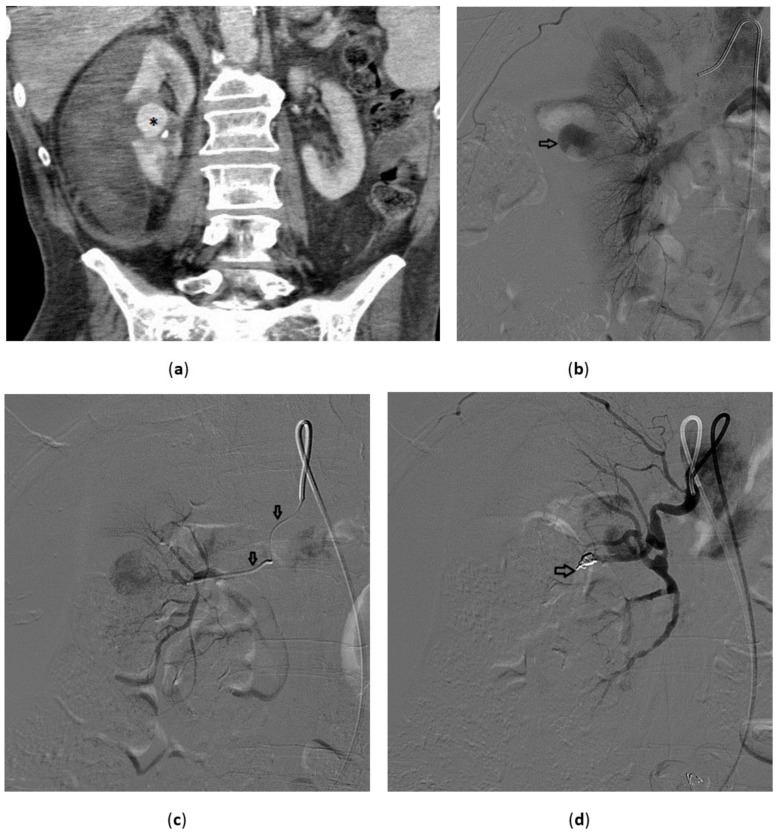
Renal pseudoaneurysm 79-year-old male with hematuria; previous attempt to insert a percutaneous nephrostomy on the right kidney. (**a**) Contrast enhanced CT: hyperdense round lesion (*) in the middle of the right kidney compatible with pseudoaneurysm with a large perirenal hematoma; (**b**) Right renal angiogram confirms the lesion (arrow). (**c**) A microcatheter was advanced in the feeding artery (arrows) and the visualization of the pseudoaneurysm after contrast media injection confirmed the correct position. (**d**) Two microcoils were deployed (arrow) and the pseudoaneurysm was no longer visible.

Diagnosis is complicated due to non-specific symptoms and official guidelines are not available. However, RAP should be suspected in case of history of any kind of renal intervention. The first line of examination should be Color Doppler Ultrasound (US). According to Cohenpour et al. [25], pseudoaneurysms may resemble a cystic mass on B-mode US. Color Doppler shows typically to-and-fro movement within the mass. Perirenal hematoma may also be present, as shown by Heye et al. [26]. Confirmation by CT-angiography is frequently needed. A well-circumscribed dense collection of contrast medium similar to arterial enhancement confirms the diagnosis. Usually there is no involvement of renal parenchyma (Figure 2); however, other signs may be present such as subcapsular or perirenal hematoma.

Treatment options for RAP include embolization, nephrectomy, and observation. Angioembolization can be effective in 96% of cases (Figure 2); the second-line treatment is nephrectomy.

Different embolic materials and strategies can be used to occlude RAPs and coils/microcoils or plugs are the devices used more frequently. In case of small RAP, coiling of the sac can be attempted, while for a large sac, occlusion of the feeding artery is the preferred solution (Figure 2). 

Chavali et al. [27] reported an incidence of RAP after partial nephrectomy from 0.5% to 1.7%, with different time of presentation. They reported a success rate of embolization of 100%, without any decline in renal function.

In their work, Ngo et al. [22] showed that in a series of 93 patients with penetrating trauma of the abdomen involving the kidney, 9.3% developed RAP, while data about RAP arising from blunt trauma are anecdotal.

### 3.3. Renal Arterovenous Fistula (AVF)

**Figure 3:**
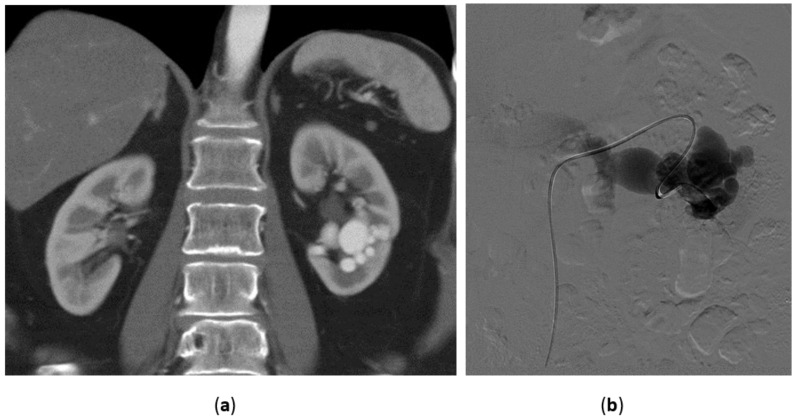

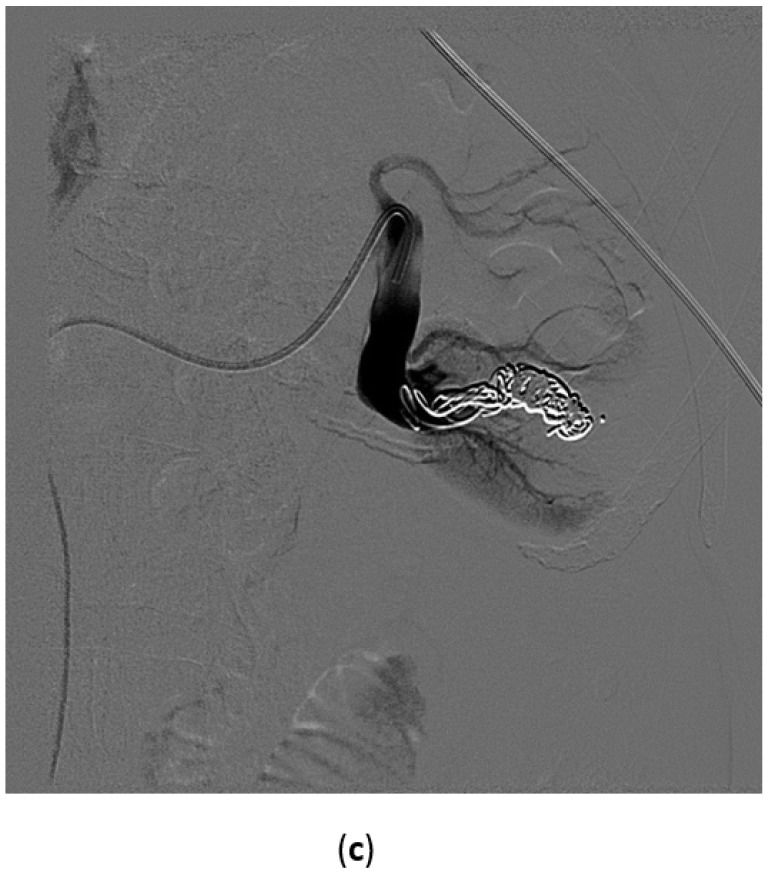
Arteriovenous fistula 59-year-old female, occasional finding on US examination. The patient had no history of previous trauma or renal intervention; thus, the AV fistula was supposed to be congenital. (**a**) Contrast-enhanced CT (arterial phase). The coronal plane well shows dilated vascular structures in the inferior half of the left kidney; (**b**) Selective angiogram of the left kidney confirms the AV fistula with huge dilatation of the renal vein; (**c**) Disappearance of the fistula after embolization with several detachable coils.

The incidence of renal AVF is variable, estimated between 0.3–1.9% in native kidneys and 6–8% in renal transplants. Approximately 70% of cases are acquired or iatrogenic, while about 20% are congenital. Most cases have an iatrogenic cause and are usually observed after renal biopsy, nephrostomy, renal traumas, neoplastic or inflammatory lesions or renal surgery. Rare cases of congenital AVFs have been described and it has been hypothesized to be congenital aneurysm already present at birth that erode into adjacent veins (Figure 3).

Since Wallace et al. [28] described the first case of successful TAE, this technique has been chosen as first modality treatment of renal AVFs. In the past, surgery, such as nephrectomy or ligation of the renal artery, were chosen to manage idiopathic and acquired types. Due to the large diameter of the fistula and the large amount of blood flow through it, the risk of coil migration during TAE has been reported, which may cause pulmonary embolism. Lately, different works have shown improvement in TAE of renal AVFs, thanks to the development of endovascular devices. Detachable balloons, steel coils with coil anchor mechanism, and the use of n-butyl-cyanoacrylate with simultaneous occlusion of efferent vein have been described as embolic techniques for this type of lesion [29]. 

### 3.4. Renal Trauma

Injury of the genitourinary tract occurs in 10% of abdominal traumas [30]. Renal trauma accounts for approximately 3% of trauma admissions, with blunt injuries being approximately 80% of the total [31], caused by street accidents or falls. However, prevalence may change based on the context, with penetration trauma (due to stabbing wounds or gunshot wounds) being more represented in urban contexts [32]. 

In addition, iatrogenic lesions may be a complication of urological procedures, such as percutaneous nephrolithotomy, renal biopsy, or partial nephrectomy, and have patterns typical of renal trauma [33].

Different urological societies have released their guidelines for the management of renal trauma and all these guidelines agree that the first evaluation must be of the patient’s hemodynamic status. If shock signs are present (such as systolic blood pressure below 90 mmHg), the patient should immediately undergo laparoscopic exploration. If the patient is stable, a conservative approach may be attempted.

The American Association for the Surgery of Trauma (AAST) has developed a five-grade evaluation scale of renal trauma, which states as follows:

1. Grade I: renal contusion, possibly with microscopic gross hematuria, or nonexpanding subcapsular hematoma without a parenchymal laceration;
2. Grade II: nonexpanding perirenal hematoma confined to renal retroperitoneum or a renal cortex laceration (<1 cm) without urinary extravasation;
3. Grade III: renal cortex laceration (>1 cm) and no urinary extravasation or collecting system rupture;
4. Grade IV: renal cortical laceration extending into the collecting system, or a segmental renal artery or vein injury (with parenchymal infarct), or main renal artery or vein injury with contained hematoma;
5. Grade V: shattered kidney avulsion of the renal pedicle, or thrombosis of the main renal artery.

In trauma patients, total body CT must be performed and for the evaluation of the abdomen unenhanced, arterial and portal phases must be acquired for a complete evaluation of the status of all parenchymal organs and for the detection of active bleeding. CT is the ideal choice for the aforementioned scoring of renal trauma.

The AAST scale can be further simplified in Low-Grade Renal Trauma (LGRT) (grade I-II) and High-Grade Renal Trauma (HGRT) (grade III to V), evaluating the extension to the urinary collecting system or to the renal pedicle. LGRT, which accounts for almost 90% of renal traumas [34], is usually treated in a conservative way with observation, bed rest, and serial blood counts.

HGRT management is complex and debatable, and every patient must be carefully evaluated.

**Figure 4:**
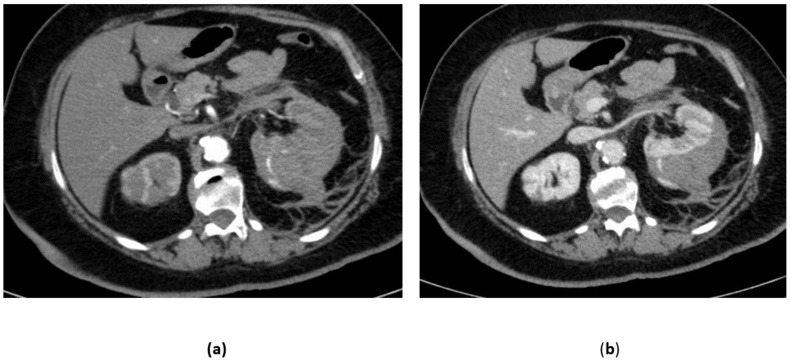

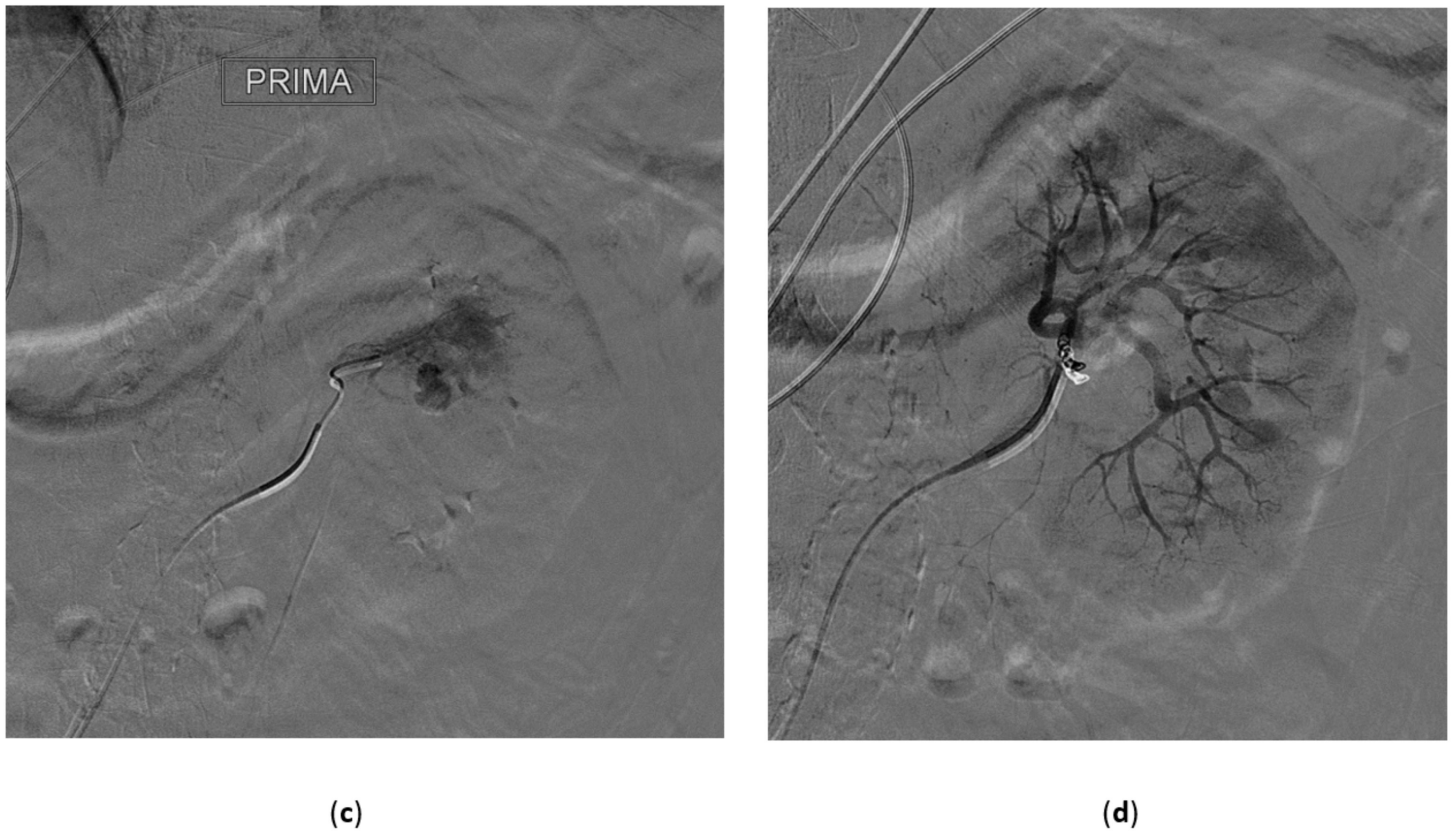
Renal trauma 59-year-old male. Car accident, hematuria. (**a**,**b**) Contrast-enhanced CT shows a left perirenal hematoma with an active bleeding inside; (**c**) Superselective angiography confirms the bleeding, which was treated by deployment of a microcoil (**d**).

Angiography with embolization has a limited role reserved for cases of renal trauma in which CT has identified an active arterial bleeding close to a renal fracture or in the context of a subcapsular or perirenal hematoma (Figure 4).

Since its introduction in 1973 by Bookstein and Ernst [35] and thanks to its progressive improvements, transcatheter embolization has become an important modality for the treatment of active renal bleeding, especially because most laparoscopy end up with a nephrectomy [36]. In recent years, even grade V renal traumas, historically treated with an open approach, are shifting towards a minimally invasive management. Several clinical criteria have been proposed to evaluate the opportunity to use renal embolization to deal with such high-grade injuries, as AAST grade, mechanism of injury, patient clinical stability and concomitant other organs injuries [37]. Surgery should be reserved in case of incontrollable hemorrhage, renal pedicle avulsion, and expanding uncontained hematoma of retroperitoneum. As stated by Ramaswamy et al. [38], renal embolization allows preventing total nephrectomy up to almost 80% of cases in case of HGRT.

Long-term results have not been so largely reported. In one series by Stewart et al. [39], 10 patients with grade V renal trauma that underwent embolization were followed for a mean time of 2 years: only one of them developed a newly diagnosed hypertension, easily controlled with medical therapy. No other major complication was reported.

As reported in the review by Muller and Rouvière [40], the technical success of renal artery embolization is frequently above 85%, even if more than one procedure may be necessary. In case of repeated failures, nephrectomy remains the gold standard treatment.

### 3.5. Renal Angiomyolipoma

Renal angiomyolipoma (AML) is a benign tumor composed of hamartomatous tissue, dysmorphic blood vessels, smooth muscle cells, and mature adipose tissue in variable proportions [41].

Several factors influence patient management: asymptomatic small (<4 cm) AML in non-pregnant patients does not need prompt intervention and active surveillance is suggested while symptomatic patients or AML >4 cm should be considered for intervention.

Even though AML are benign tumors, they may grow substantially, and as they grow, they often become more vascular, developing aneurysms and tortuous vessels, which have a high probability of rupture [42,43,44]. As AMLs grow in size, bleeding risk increases; furthermore Yamakado et al. observed that AML, which contains aneurysms larger than 5 mm, have a greater probability for predicting bleeding than renal AML size [42,45].

In the past, surgery, such as nephrectomy or partial nephrectomy, was the first line treatment of AML. In recent years, less-invasive techniques have increased with the use of laparoscopic approaches. However, the development of non-surgical therapies has changed the approach to patient care in many cases, also in consideration of the morbidity and possible serious complications associated with surgery [46].

**Figure 5:**
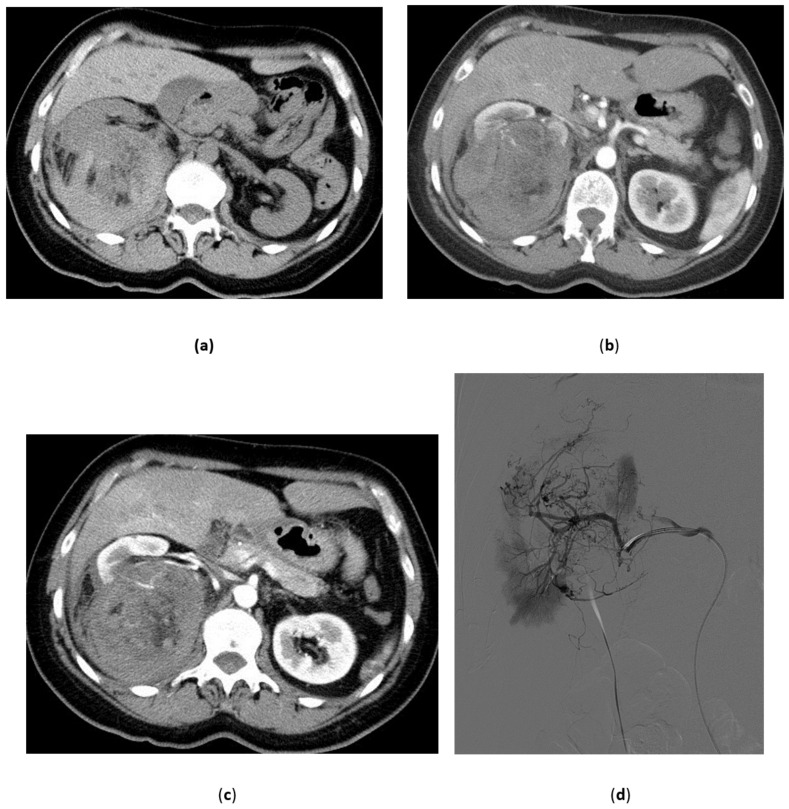

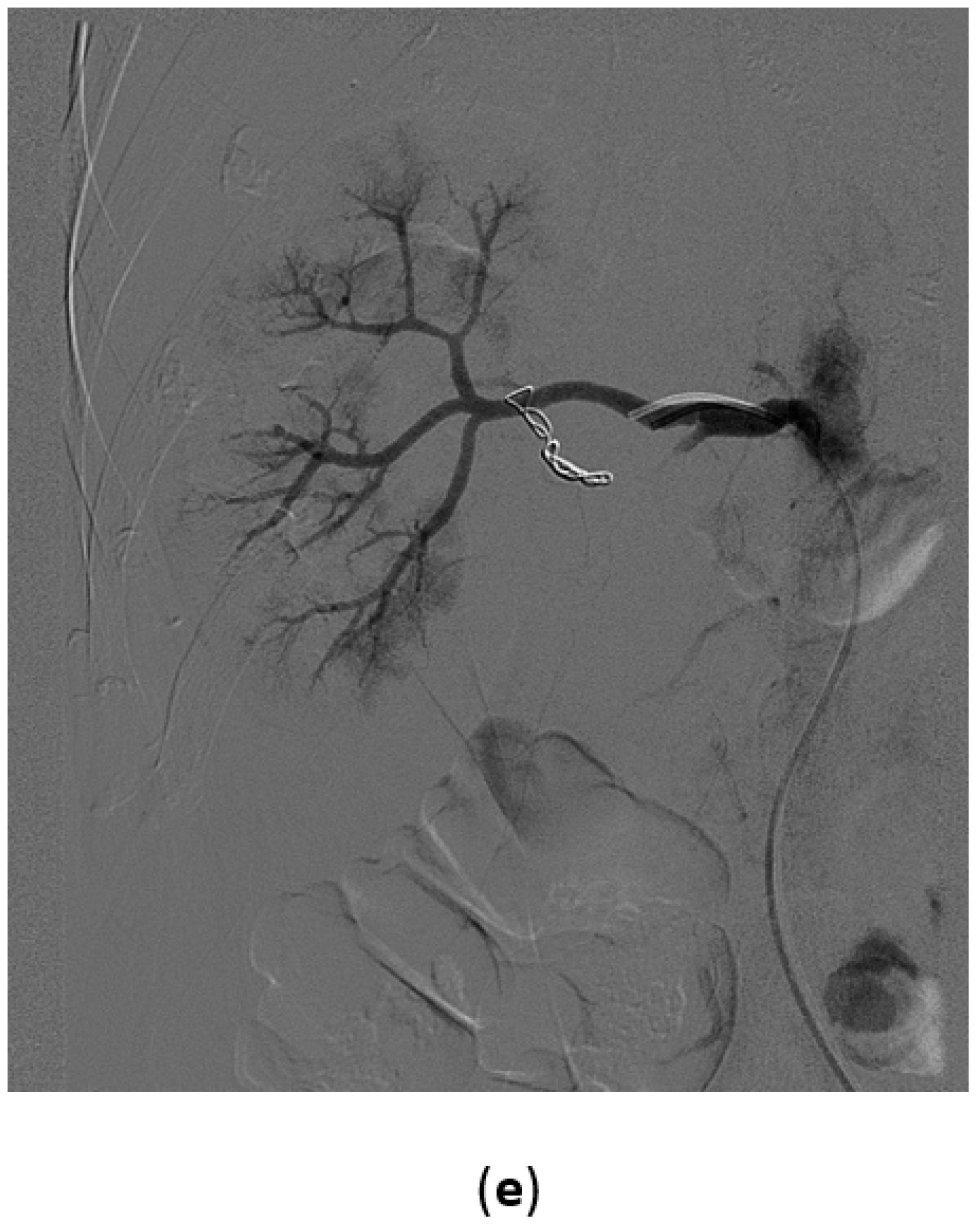
Angiomyolipoma. 54-year-old female with sudden onset of right flank pain. (**a**) Unenhanced CT: the right kidney is dislodged anteriorly by a perirenal hematoma with a mixed pattern consisting of fat attenuation density and high attenuation density due to acute bleeding; (**b**,**c**) Contrast enhanced CT: AML shows a “jet” sign due to active extravasation inside the mass; (**d**) Selective angiogram of the feeding artery confirms the typical aspects of AML: rich vascularization with aneurysmatic aspects; (**e**) Complete devascularization of the AML after embolization with particles and microcoils.

Currently, a popular management of renal AML is transcatheter arterial embolization (TAE), as it saves normal renal parenchyma, manages acute tumor bleeding, and can also be used as a prophylactic treatment before surgery to reduce blood loss during the intervention [41,47] (Figure 5).

In literature, greater safety or efficacy of a specific embolic agent has not been reported, but it has been demonstrated that embolization with glue or particles, such as PVA, should be favored, due to their capability of occluding small arteries supplying the AML [48,49,50]. Coils lack the capability of small-vessel embolization and they determine the inability to re-embolize if the AML keeps growing or recurs, therefore they should be avoided. Additionally, Villalta et al. compared the use of embolic particles smaller and greater than 150 µm in a non-randomized study and observed that patients who underwent embolization with smaller agents were nearly six times more likely to require an additional embolization, favoring the use of larger particles if these are selected as the embolic agent [51]. Furthermore, they reported that there was an increased occurrence of pulmonary complications (due to migration of embolic material in pulmonary arteries) in patients in which smaller particles were used. This can be explained by the fact that AMLs are made up of aberrant blood vessels, which may provide a direct channel to the venous circulation, allowing access to the systemic circulation and embolization to distant sites, even in the absence of appreciable arteriovenous shunting during angiography.

Furthermore, embolization is effective also to induce tumor shrinkage [52]. However, the authors found that high-fat content lesions (defined as having greater than 50% fat content), while still showing 50% reduction in volume, are less responsive to embolization than low-fat content lesions which decreased by 84%. This study also found that the greatest reduction in tumor size occurs early after embolization before gradually plateauing.

A possible complication that has been reported which can occur in the days following the embolization of renal AMLs is postembolization syndrome; pain, nausea, fever, and leukocytosis characterize this condition, which is usually self-limited and resolves with standard supportive care. Even in patients with pre-existing renal insufficiency, renal function is often unaffected after embolization. Other possible rare complications are represented by non-target embolization (normal renal parenchyma or other organs), abscess formation which may require drainage, and vascular injuries such as renal artery rupture or dissection. Overall, embolization is a well-tolerated procedure [53].

### 3.6. Malignant Renal Tumors

Embolization for malignant renal tumors as clear cell carcinoma has two main indications: pre-operative embolization and palliative embolization.

The first indication, pre-operative embolization is a matter of debate. The possible advantages are to decrease blood loss during nephrectomy and to improve cleavage of the lesion, thanks to the inflammatory reaction in the embolized renal parenchyma [54]. This procedure can be considered mainly for large lesions, with rich vascularization, seen on CT, because it has been postulated that embolization may improve the surgical approach to the vascular pedicle in stage III tumors with involvement of the renal vein by collapsing the perihilar vascular network. Furthermore embolization provides a better cleavage plane in cases with renal capsule involvement [55]. In this setting, in order to achieve a more effective devascularization of the tumor to be excised, liquid embolic materials, such as ethanol or glues, can be used but also not-resorbable embolic particles are frequently employed; resorbable embolic agents are not suggested because of their temporary effect.

**Figure 6:**
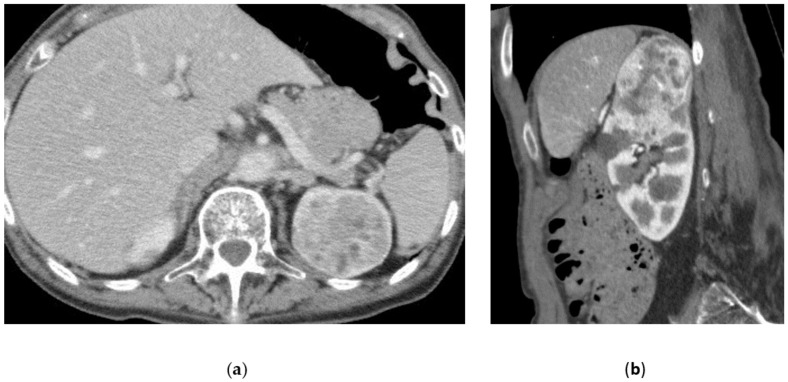

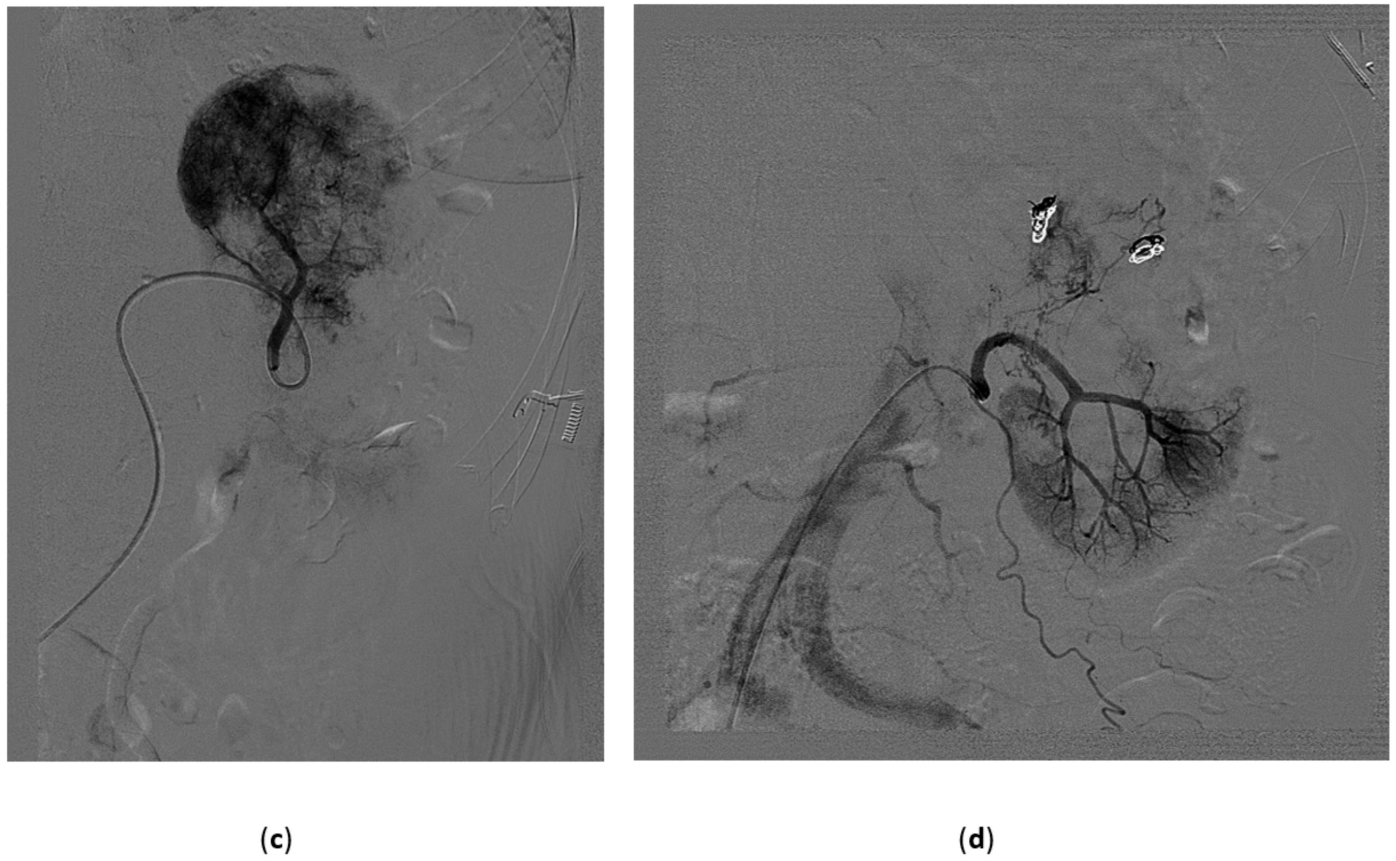
Malignant renal tumor. 91-year-old female with hematuria, anemia, and left flank pain. Palliative embolization of the left kidney was scheduled. (**a**,**b**) Contrast-enhanced CT (axial and sagittal plane) shows a huge not homogeneous mass of the superior half of the left kidney; (**c**) Selective angiography: rich vascularized renal lesion. (**d**) Angiogram (after embolization): the lesion is no longer visible; the inferior pole of the left kidney shows normal aspect.

Concerning palliative embolization, no controversy exists about its role in patients with advanced renal cancer [55]. Embolization may help relieve symptoms such as low back pain resistant to analgesic therapy or other less-frequent symptoms such as arterial hypertension, hypercalcemia, and polycythemia and for treatment of complications such as persistent macroscopic hematuria with anemia. In this context, embolization can be tailored, and procedure focused mainly to the arteries feeding the tumor in order to reduce drawbacks of embolization as post-embolization syndrome, infections, and deterioration of renal function. Surgery is much more aggressive than a “tailored” embolization (Figure 6).

Palliative embolization in patients with end-stage renal disease has shown an improvement in survival of several months [56].

## 4. Bladder

Intractable bladder hemorrhage is a potentially life-threatening occurrence that may complicate a number of neoplastic and inflammatory conditions in the pelvis, including unresectable bladder cancer, radiation-induced hemorrhagic cystitis, and cyclophosphamide-induced hemorrhagic cystitis [57]. Bladder cancer often causes recurrent refractory hematuria due to sloughing of tumor mass. In hemorrhagic cystitis, an initial acute inflammatory response with tissue edema is followed by neo-angiogenesis of thin, telangiectatic mucosal vessels, which tends to bleed and represent a potential source of severe blood loss [1,2].

Intractable hematuria raises major therapeutic problems because of the advanced disease, generally poor condition of the patients and lack of consisting evidence for the efficacy of any treatment. Conservative therapies include bladder irrigation with alum solution, instillation with formalin or silver nitrate, Helmstein balloon compression, cystoscopy with clot evacuation and endoscopic diathermy [1,2,57]. When the hematuria persists despite conventional treatments, the management may become difficult. Prolonged or repeat hospitalization for bladder irrigation and multiple blood transfusion are not practical; surgical options include internal iliac artery ligation and urinary diversion, with or without cystectomy, but these procedures are associated with an unacceptably high morbidity and mortality in this population of patients [57].

In this setting, superselective transcatheter embolization of the vesical arteries appears as an attractive therapeutic option, being a minimally invasive procedure that allows an immediate control of severe bleeding, minimizing complications, as well as a sustained control of hematuria, allowing the patient to stay at home without catheters.

**Figure 7:**
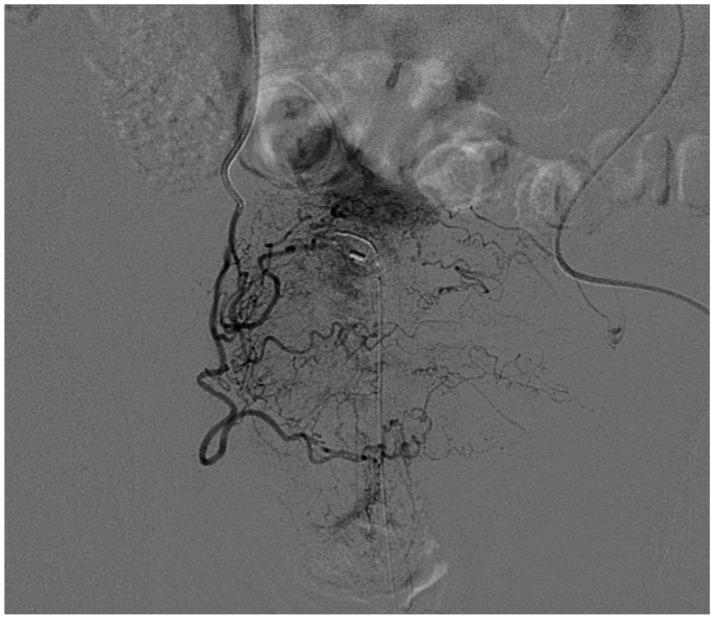
Angiography of vesical arteries. 77-year-old male, prostatic cancer treated with radiotherapy, macroscopic hematuria due to radiation cystitis. Superselective catheterization of the right vesical arteries shows abnormally hypertrophic vessels.

Usual findings at angiography include abnormal hypervascularity of the vesical arteries or even a mass, but contrast extravasation is not commonly seen (Figure 7).

**Figure 8:**
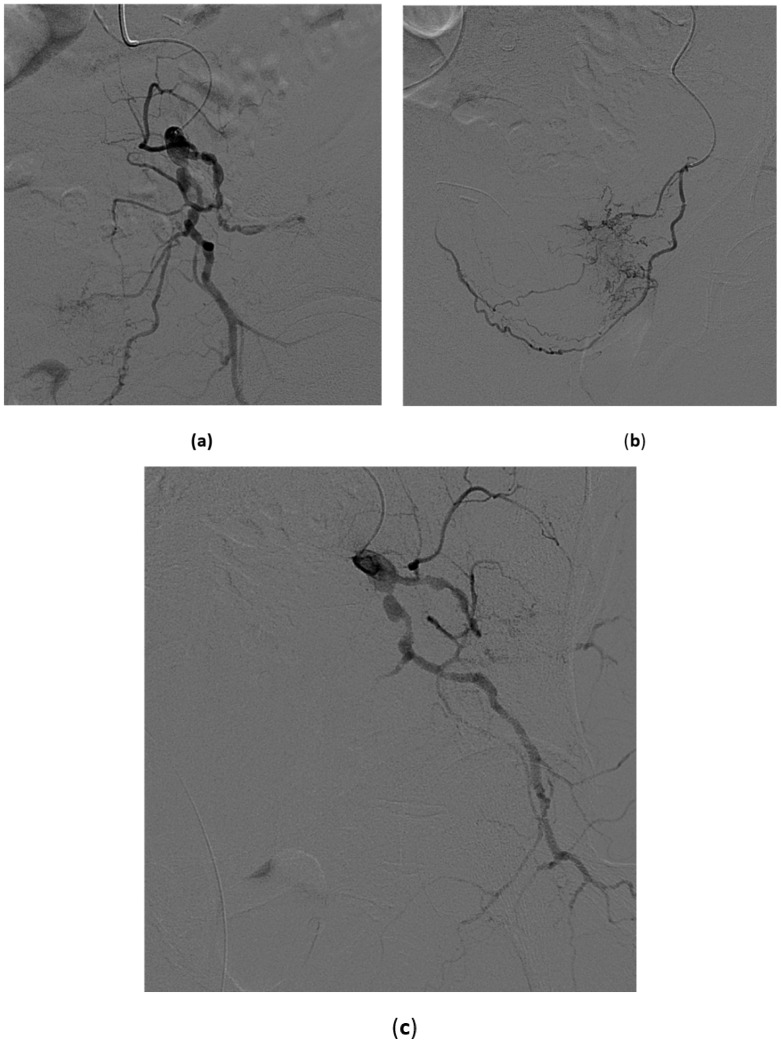
Superselective embolization of the vesical arteries. 104-year-old male, advanced bladder cancer with macroscopic hematuria. (**a**) The preliminary angiogram shows the vesical arteries originating as branches of the anterior division of the internal iliac artery. Diffuse atherosclerotic irregularities of the vessels are present; (**b**) Superselective catheterization of the vesical vessels is performed and an abnormal hypervascularity is demonstrated; (**c**) After embolization with 500–700 µm particles, the final angiogram shows adequate occlusion of the vessels. The procedure was then repeated on the contralateral side.

According to the delineated anatomy and the possibility to perform a superselective catheterization of the vesical branches, three main techniques of embolization have been described [58,59,60,61,62]:

- Superselective catheterization of vesical arteries is the preferred approach as it allows a more targeted delivery of the embolizing particles, thus reducing to a minimum the risk of side-effects from non-target embolization. A coaxial microcatheter is used for selective catheterization. Polyvinyl alcohol particles or tris-acryl gelatin microspheres particles are then released, mixed with contrast medium to make them radiopaque, and detectable with fluoroscopy during delivery. As the most distal branches are embolized, larger particles are injected (Figure 8). In cases in which contrast extravasation is identified, embolization of the feeding artery with n-butyl-2-cyanoacrylate glue mixed with lipiodol has been described [59].

- The coil blockade technique is the alternative approach when the vesical arteries cannot be selectively catheterized. It consists of using 0.018-inch microcoils or a vascular microplug of the appropriate length and diameter to occlude a distal branch at its ostium. Then, embolizing particles are released. In this way the distal branch is protected from unwanted embolization and the particles tend to flow selectively into the vesical vessels, also because of the lower peripheral resistance of bleeding vessels.
- When the catheterization of the main distal branches of the anterior division of the hypogastric artery is not possible, the catheter tip is left in the anterior division and proximal embolization is performed using 0.035-inch coils of adequate length and diameter or mechanically disrupted absorbable gelatin sponge powder sheet.

The embolization should be as distal as possible, using non-absorbable particles, and both sides should be treated [7,59,61]. Use of various embolic materials have been reported over the years, including particles, coils, gelatin foam, gelatin sponge and n-butyl-2-cyanoacrylate. Although the efficacy of the different embolizing agents have not been directly compared, non-absorbable particles should be preferred because of the high success and low complication rates [7,58,59]. When absorbable gelatin sponge particles are used, recanalization of the vessels may develop after 2 to 3 weeks [63]. Moreover, the embolization should always be bilateral because a higher risk of recurrence has been reported after unilateral procedures, probably due to the rich collateral blood supply to the internal iliac artery from the contralateral internal iliac, inferior mesenteric, external iliac and femoral arteries [7,60,64]. The partial physiological alterations in pulse volume, blood flow and mean pressure avert the risk of ischemic infarction [65].

### Results of the Procedure

Embolization of the internal iliac artery to control intractable massive hematuria was initially reported by Hald and Mygind in 1974 [66], and since then, the indications for transcatheter arterial embolization in uncontrollable bleedings have increased markedly, with better outcomes and lower complication rates, thanks to the refinement of both techniques and instruments. In particular, the introduction of microcatheters has allowed a more selective catheterization of distal vessels, with higher success rates and lower side effects.

The current literature consists mainly of observational studies and case reports; there are no randomized studies or control groups for comparison with other treatments. From current studies, trans-arterial superselective embolization of vesical arteries appears to be a safe and effective treatment to manage intractable hematuria and to provide sustained bleeding control in a number of conditions, including bladder cancer and hemorrhagic cystitis [1,59,62,67]. It should be considered as an alternative, less-invasive palliative treatment for those patients in whom conservative therapies fail and, in selected cases, it may be considered as the treatment of choice to obviate the need for surgery in severely ill patients [1,61,62,68].

A recent systematic review by Taha et al. [67] included studies published from 1978 to 2016 for a total of 295 patients embolized for intractable bladder hemorrhage secondary to bladder cancer, prostate cancer, radiation cystitis, cyclophosphamide-induced cystitis or severe infection, and report an overall success rate of the procedure from 43% up to 100%. In the last few years, the possibility of superselective embolization has increased the success rate of the procedure, with reported success in 90% of patients when the vesical arteries can be identified [68]. Higher recurrence rates have been described for patients with radiation cystitis even after bilateral embolization, suggesting that this population has a higher risk of rebleeding and repeat embolization may be required [59]. The longer survival of these patients may also contribute to the higher recurrence rate observed.

Follow-up after embolization is usually short and mortality high because treated patients are mostly elderly and with advanced malignancies; however, mortality is rarely due to re-bleeding [59,67]. In a study including 44 patients, Liguori et al. reported a permanent control of bleeding in 43% of cases at a mean follow-up of 10,5 (1–97) months. A second embolization was necessary in five patients and was successful in two of them [61]. Pisco et al., in a large study including 108 patients treated with embolization of the anterior division of the hypogastric artery for uncontrollable hemorrhage secondary to pelvic neoplasms, reported complete control of bleeding in 73% of cases at 6 months [7]. Some smaller studies also reported successful results in the long-term after embolization for severe hematuria due to malignancies [64,69].

Side-effects reported in the literature include buttock pain, perineal pain, bladder necrosis, Brown-Sequard’s syndrome, gluteal paresis, and skin necrosis [6,7,70,71,72,73]. However, most of these complications occurred when vesical arteries were not selectively catheterized and the reported ischemic complication rate was high, up to 68.5% [7]. In more recent studies in which superselective embolization was used complication rates are around 10% and are usually minor and self-limiting, such as gluteal pain, fever, nausea, and vomiting [59,60,67]. When a superselective catheterization is not possible, the embolization should be as distal as possible in the anterior division of the internal iliac artery in order to spare the gluteal artery and avoid complications such as severe buttocks and upper thighs pain.

## 5. Penis

Endovascular interventional procedures for the penis are indicated only for the treatment of “priapism”. The term “priapism” defines a condition of partial or complete penile tumescence that lasts for more than 4 h beyond a sexual stimulus or is not related to sexual stimulus [3] although other authors consider 6 h as lasting time [74]. Priapism is a complex medical emergency with different pathophysiology, which may require fast medical and surgical acts to avoid complications such as irreversible erectile dysfunction [75].

For clinical management, priapism can be distinguished into two groups: low-flow priapism, usually ischemic (with a veno-occlusive mechanism) and high-flow priapism, usually non-ischemic (with an arterial mechanism) [75]. We will focus on this last condition.

### 5.1. High-Flow Priapism

High-flow priapism is defined as a persistent erection caused by unregulated cavernous arterial inflow. The first description was done by Burt et al. in 1960 in a man who developed a persistent erection after a traumatic coitus [76]. The high-flow etiology of priapism is less frequent than the low-flow etiology, and trauma is the most common cause. Frequently these lesions depend on injuries to the crura or corpora, which cause a tear to the cavernous artery or one of its branches, resulting in a fistula or a pseudo-aneurysm and an unrestrained arterial flow to penile sinusoidal spaces. Usually mechanisms responsible of penile injuries are coital traumas, pelvic fractures, kicks to the penis or perineal region, iatrogenic penile lesions, (complications of penile invasive diagnostic procedures or vascular lesions complicating surgical interventions), or metastatic infiltration of the corpora [77]. Furthermore, high-flow priapism can develop subtly because the arterio-cavernosal fistula arises several days after a perineal or genital trauma. This delayed manifestation is explained with resolution of the spasm of the damaged penile artery or an alternative mechanism can be the recanalization of a penile artery previously occluded by a clot [74].

Any tear of a cavernous artery or of one of its branches increases the pooling of blood in the sinusoidal space with consequence erection; however, no ischemic changes to cavernous tissues are observed because arterial blood flow is maintained. The patient does not feel pain during erection, which is more a tumescence rather than a rigid erection [3]. High-flow priapism, as opposed to low-flow priapism, is not considered to be an emergency and immediate treatment is not required.

### 5.2. Clinical, Laboratory, and Imaging Findings

History of a previous trauma, the absence of pain associated with the erection, and the prolonged duration of erection without any progressive discomfort are typical signs of high-flow priapism. Physical examination in these patients show an engorged or only partially erect penis [78]. Sometimes, at palpation, there may be some tenderness and a residual bruising. Blood gas analysis of corpora is useful to differentiate between high-flow from low-flow priapism: in the first condition, pO2 is superior to 90 mmHg, while in low-flow priapism, pO2 is inferior to 40 mmHg.

In addition, Color Doppler ultrasound (CDU) can be helpful and must be performed using a high-frequency transducer: the patient stands with the legs in the frog position with the scrotum elevated, the cavernosal arteries are visualized from their origin in the perineum along the ventral aspect of the penile shaft. In high-flow priapism, CDU demonstrates a “low-resistance, high-velocity” arterial waveform [79], while in low-flow priapism, cavernosal arterial flow typically appears as a “high-resistance, low-velocity” waveform without any arterial flow aspect. The sensitivity of CDU in localizing an arteriocavernosal fistula is reported to be around 100%. Furthermore, on gray-scale ultrasonography, the arteriocavernosal fistula can be detected as a hypoechoic area surrounded by echogenic tissue.

### 5.3. Treatment of High-Flow Priapism

High-flow priapism does not result in ischemia within the corpora cavernosa and for this reason, it should be managed as an emergency. The first-line treatment is conservative management because this strategy has a 60% chance of spontaneous resolution [80]. However, Savoca et al. [81] underlined that long-standing, high-flow priapism can lead to cavernosal fibrotic changes and erectile dysfunction; therefore, more aggressive treatment must be considered for patients not responding to conservative management. Conservative management can be done simply using manual compression. As an alternative, ice application can be attempted, with the aim of inducing vasospasm of the penile artery with clot formation and occlusion of the fistula. Another strategy consists of the ultrasound-guided compression technique: this is particularly helpful when a cavernosal pseudoaneurysm is identified on CDU. The technique consists of a prolonged compression done with the CDU probe in “real-time” observation of the fistula until it disappears [82].

If conservative management is not successful, selective embolization of the cavernosal artery can be attempted. With this technique, which was described for the first time by Wear et al. in 1977, a temporary occlusion of the cavernosal artery by means of embolic materials is obtained. In this way a cicatricial closure of the fistula and delayed rechanneling of the embolized artery can be observed [83]. This procedure is not free from complications and residual erectile dysfunction or recurrence after embolization treatment has been reported in approximately 15 to 22% of treated patients [81,84].

**Figure 9:**
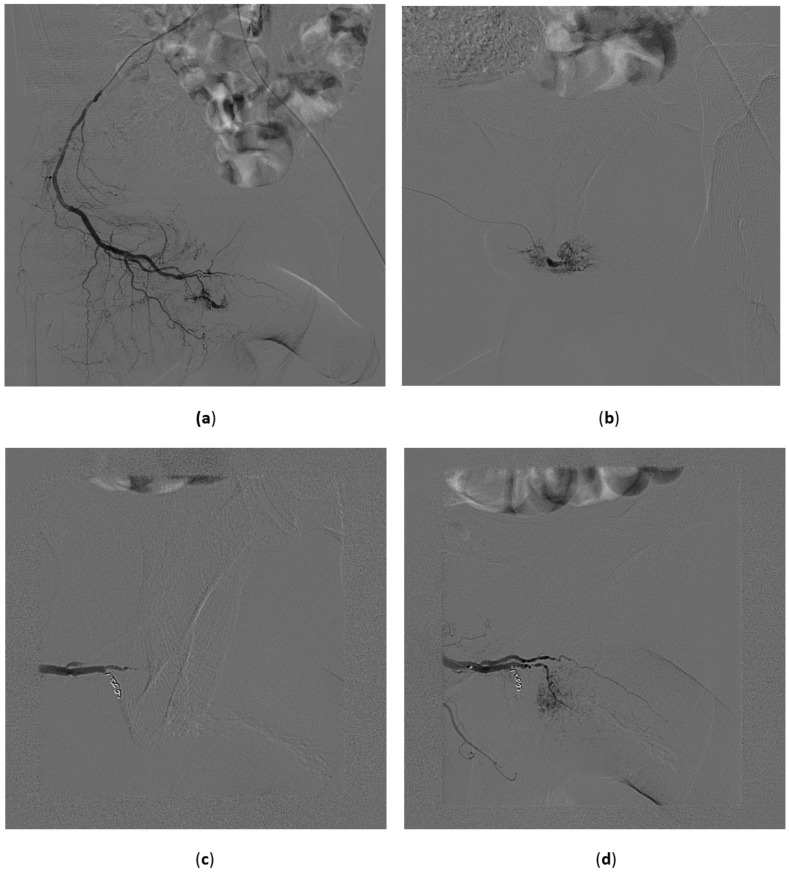
High-flow priapism. 45-year-old male, recent perineal trauma. CDUS reveals an arterocavernous fistula on the right side. (**a**) Selective angiography with injection in the proximal pudendal artery shows the cavernosal fistula at the basis of the penis; (**b**,**c**) A microcatheter was advanced with the tip to the site of the fistula and a small microcoil was deployed with complete occlusion of the lesion (**c**,**d**).

In our experience, from a technical point of view, procedures are performed with a femoral unilateral approach. A pelvic angiogram with contrast media injection in the distal aorta can be useful for identifying from which side the feeding artery fills the cavernous fistula or pseudoaneurysm. The lesions are unilateral, but bilateral lesions are not rare. After catheterization of the internal iliac artery (with conventional 4 or 5 Fr catheters), the use of a microcatheter is mandatory in order to achieve embolization as distal as possible (Figure 9).

Considering that pudendal and penile arteries tend to increase their caliber due to high-flow priapism, microcatheter navigation appears feasible in almost all cases. Various embolic materials can be used for this procedure, the first one, used in the early days, was autologous blood clot, while currently “ready for use” materials such as gelatin sponge, microcoils, polyvinyl alcohol, and glue are preferred [85,86,87].

All these embolic materials achieve a similar 75% technical success rate. In our experience, we used either microparticles or microcoils (Figure 9). The latter was preferred in cases where large vessels were injured, while particles were chosen in cases where the lesion appeared as a small fistula. No vasospasm was observed and no pharmacological agent was adopted to prevent it.

Only in cases where embolization fails, we resort to surgical treatment of high-flow priapism, which consists of transcorporal fistula ligation, as it carries a higher risk of future erectile dysfunction. Surgery management complications include penile gangrene, gluteal ischemia, purulent cavernositis, perineal abscess, and erectile dysfunction (as high as 50%) [88].

## 6. Conclusions

Interventional radiology provides effective and minimally invasive treatment options for multiple vascular, neoplastic, inflammatory, and traumatic conditions of the urogenital system. These procedures should always be considered when evaluating the best therapeutic approach for each patient.

## Data Availability

Not applicable.
